# Management of solid and sub-solid lung nodules

**DOI:** 10.1186/1470-7330-14-S1-O24

**Published:** 2014-10-09

**Authors:** Christian J Herold

**Affiliations:** 1 Department of Radiology, University of Vienna, Austria

## Introduction

Pulmonary nodules present a unique challenge for radiologists which includes the detection of malignancy and identification of benign disease, recommending the appropriate procedures to avoid unnecessary thoracotomy, and ensuring that excessive radiation from follow-up computed tomography (CT) examinations is kept to a minimum. Although seasoned radiologists can use an approach based on their extensive experience, in this era of evidence-based medicine, guidelines have been established that include both radiologic and clinical factors in an integrated fashion. Such guidelines have been developed by the Fleischner Society, first in 2005, for the management of small pulmonary nodules detected on CT scans, and then, in 2013, for the management of sub-solid nodules [1].

For incidentally detected solitary nodules, these guidelines integrate elements as individual risk factors (via pre-test probability), with lesion size, growth rate, and morphology, based on imaging characteristics. Relevant risk factors to consider in the evaluation of pulmonary nodules are smoking history and patient age. Patients with a substantial smoking history and over 40 years of age are defined as high-risk patients, whereas low-risk patients are those with a negligible smoking history and no other known risk factors, and who are below 40 years of age.

Based on the Fleischner Society recommendation [1], lesions less than 4 mm do not require any further invasive or non-invasive measures or evaluations. However, lesions with a diameter of more than 8mm should be evaluated with either follow-up CT scans, including dynamic, contrast-enhanced scans, positron emission tomography (PET)-CT, and biopsy. For lesions that fall between the 4 mm and 8 mm size, CT follow-up with a limited number of scans is recommended.

Sub-solid nodules are often found during screening studies or incidentally on CT scans. They are characterized by their appearance as rounded areas of increased attenuation, and a homogeneous or heterogeneous texture. Sub-solid nodules can be subdivided according to their composition: non-solid lesions that consist of ground-glass opacity only; and part solid lesions that contain both ground-glass and soft tissue elements.

Sub-solid nodules can regress, persist, or grow. Small persistent non-solid nodules are often atypical adenomatous hypoplasia (AAH) and focal fibrosis, whereas non-solid and part-solid nodules that increase in size typically indicate malignancy, which can include adenocarcinoma in situ (AIS), minimally invasive adenocarcinoma (MIA), and invasive adenocarcinoma, as well as lympho-proliferative disease. How these lesions develop has not been entirely elucidated as yet, but it seems that a small percentage of ground-glass opacities (AAH) develop into larger lesions that can eventually comprise solid components due to invasion and alveolar collapse [2]. In any case, radiologically, an increase in lesion density, growth, or the development of solid portions are all indicative of malignancy.

CT analysis and surveillance of sub-solid nodules is the modality of choice for the identification of malignant lesions. The primary factor is nodule size. While lesions less than 5 mm in diameter are usually AAHs, lesions greater than 15 mm is size are malignant, and are typically MIAs and invasive adenocarcinomas. Generally, a diameter of more than 8 mm is indicative of malignancy. Another important indicator of malignancy is lesion growth. The growth of the lesion may affect either the ground-glass or the solid components, or both. Volume doubling times of AIS and MIAs are much longer, compared to invasive adenocarcinomas and squamous cell lesions.

Morphologically, the presence of lobulated borders, air bronchograms and bubble-like lucencies, and the development of solid portions in pure ground-glass abnormalities are all indicative of malignancy. Lesion morphology is closely related to prognosis. Nodules with an increasing percentage of solid components represent a poorer prognosis, but, for patients with pure ground-glass abnormalities or those with only a small area of a solid component, the survival rate after lesion resection is 100%.

The Fleischner Society management guidelines [2] for sub-solid lesions recommend that part solid nodules, especially those that persist or grow in size, mandate further evaluation and monitoring, and typically, require surgical resection. Smaller lesions, with a negligible area of solid components, can be managed with follow-up CT scans.

## References

[B1] MacMahon H Austin JH Gamsu G Guidelines for management of small pulmonary nodules detected on CT scans: a statement from the Fleischner Society Radiology 2005 237 2 395 400 10.1148/radiol.2372041887 16244247

[B2] Naidich DP Bankier A MacMahon H Recommendations for the Management of Subsolid Pulmonary Nodules Detected at CT Radiology 2013 266 304 317 10.1148/radiol.12120628 23070270

